# A Dual-Feature Framework for Enhanced Diagnosis of Myeloproliferative Neoplasm Subtypes Using Artificial Intelligence

**DOI:** 10.3390/bioengineering12060623

**Published:** 2025-06-07

**Authors:** Amna Bamaqa, N. S. Labeeb, Eman M. El-Gendy, Hani M. Ibrahim, Mohamed Farsi, Hossam Magdy Balaha, Mahmoud Badawy, Mostafa A. Elhosseini

**Affiliations:** 1 Department of Computer Science and Information, Applied College, Taibah University, Madinah 42353, Saudi Arabia; abamaqa@taibahu.edu.sa (A.B.); nlabeeb@taibahu.edu.sa (N.S.L.); engbadawy@mans.edu.eg (M.B.); 2 Mathematics Department, Faculty of Science, Helwan University, Cairo 11795, Egypt; 3 Computers and Control Systems Engineering Department, Faculty of Engineering, Mansoura University, Mansoura 35516, Egypt; eman_elgendy@mans.edu.eg (E.M.E.-G.); hmbala01@louisville.edu (H.M.B.); 4 Department of Information Systems, College of Computer Science and Engineering, Taibah University, Yanbu 46421, Saudi Arabia; hanimia78@yahoo.com (H.M.I.); mafarsi@taibahu.edu.sa (M.F.); 5 Mathematics & Computer Science Department, Faculty of Science, Menofia University, Shebin El Koum 32511, Egypt; 6 Bioengineering Department, J. B. Speed School of Engineering, University of Louisville, Louisville, KY 40292, USA

**Keywords:** myeloproliferative neoplasms, Philadelphia chromosome-negative (ph-negative) myeloproliferative neoplasms, deep learning (dl), morphological and textural features, vision transformers, automatic feature extraction

## Abstract

Myeloproliferative neoplasms, particularly the Philadelphia chromosome-negative (Ph-negative) subtypes such as essential thrombocythemia, polycythemia vera, and primary myelofibrosis, present diagnostic challenges due to overlapping morphological features and clinical heterogeneity. Traditional diagnostic approaches, including imaging and histopathological analysis, are often limited by interobserver variability, delayed diagnosis, and subjective interpretations. To address these limitations, we propose a novel framework that integrates handcrafted and automatic feature extraction techniques for improved classification of Ph-negative myeloproliferative neoplasms. Handcrafted features capture interpretable morphological and textural characteristics. In contrast, automatic features utilize deep learning models to identify complex patterns in histopathological images. The extracted features were used to train machine learning models, with hyperparameter optimization performed using Optuna. Our framework achieved high performance across multiple metrics, including precision, recall, F1 score, accuracy, specificity, and weighted average. The concatenated probabilities, which combine both feature types, demonstrated the highest mean weighted average of 0.9969, surpassing the individual performances of handcrafted (0.9765) and embedded features (0.9686). Statistical analysis confirmed the robustness and reliability of the results. However, challenges remain in assuming normal distributions for certain feature types. This study highlights the potential of combining domain-specific knowledge with data-driven approaches to enhance diagnostic accuracy and support clinical decision-making.

## 1. Introduction

Myeloproliferative neoplasms are a group of rare blood cancers that primarily affect the bone marrow, an organ crucial for the production of blood cells [1]. These disorders disrupt normal hematopoiesis, leading to the overproduction of one or more types of blood cells: red blood cells, white blood cells, or platelets [2,3]. The affected organs, particularly the bone marrow and spleen, experience significant pathological changes, including fibrosis, hypercellularity, and extramedullary hematopoiesis, which can severely impact patient health [4,5].

Among myeloproliferative neoplasms, the Philadelphia chromosome-negative (Ph-negative) subtypes (essential thrombocythemia (ET), polycythemia vera (PV), and primary myelofibrosis (MF) [6]) are the most common, accounting for approximately 90% of all myeloproliferative neoplasm cases [7]. In the United States, the incidence of Ph-negative myeloproliferative neoplasms has been estimated at 6–10 cases per 100,000 individuals annually over the past five years, with PV being the most prevalent subtype, followed by ET and MF [8,9,10].

The diagnosis of myeloproliferative neoplasms remains challenging because of their heterogeneous clinical presentation and overlapping morphological features [11]. Current diagnostic approaches rely heavily on a combination of clinical evaluation, laboratory tests, and imaging modalities such as X-ray, computed tomography (CT), magnetic resonance imaging (MRI), and ultrasound. While these imaging techniques provide valuable insights into organ involvement and disease progression, they often lack the specificity required to differentiate between myeloproliferative neoplasm subtypes [12,13].

For example, MRI is effective in assessing bone marrow composition and detecting fibrosis. Still, it cannot reliably distinguish between ET, PV, and MF without additional histopathological analysis (as highlighted by Wong and Pozdnyakova [14]). Similarly, ultrasound is commonly used to evaluate splenomegaly, a frequent complication of myeloproliferative neoplasms. Still, it provides limited information about bone marrow morphology.

Despite the utility of traditional imaging modalities, their limitations (such as interobserver variability, dependency on expert interpretation, and delayed diagnosis) underscore the need for automated systems. Artificial intelligence (AI) has emerged as a transformative tool in medical imaging, offering solutions to these challenges.

Recent advancements in AI-based approaches have demonstrated promising results in automating myeloproliferative neoplasm diagnosis. For example, Sirinukunwattana et al. [15] achieved significant improvements in classifying megakaryocyte morphologies via ML algorithms, highlighting the potential of AI to increase diagnostic precision. The review presented by Srisuwananukorn et al. [16] discussed the use of AI not only for the diagnosis and prognosis of myeloproliferative neoplasm but also for the automatic segmentation of abdominal CT images.

In this study, we propose a novel framework that integrates both handcrafted and automatic feature extraction techniques to classify Philadelphia chromosome-negative myeloproliferative neoplasms. This approach utilizes the strengths of traditional morphological and textural analysis alongside advanced deep learning models, enabling a comprehensive evaluation of histopathology images. The proposed methodology addresses the limitations of existing diagnostic tools by providing an automated, reproducible, and objective system for distinguishing between myeloproliferative neoplasm subtypes such as essential thrombocythemia (ET), polycythemia vera (PV), and primary myelofibrosis (MF). By combining domain-specific knowledge with data-driven representations, the framework aims to enhance diagnostic accuracy and support clinical decision-making.

The current study aims to develop a robust classification pipeline for Ph-negative myeloproliferative neoplasms via histopathology images. Specifically, we focus on extracting meaningful features from nuclei and tissue patterns, employing both interpretable handcrafted methods and state-of-the-art vision transformers such as H-optimus-0. Therefore, the objective of this study is to evaluate the effectiveness of integrating these two feature types in improving classification performance while ensuring interpretability and scalability. The following points provide a summary of the present study’s contributions:

- Development of a systematic pipeline for handcrafted feature extraction, including the statistical, textural, and spatial characteristics of nuclei;
- Utilization of H-optimus-0, a pretrained vision transformer, to capture complex histopathological patterns automatically;
- Integration of concatenated probabilities derived from handcrafted and embedded features to achieve superior classification accuracy;
- Comprehensive statistical analysis to validate the robustness and reliability of the proposed framework.

The rest of the paper is organized as follows: Section 2 provides an overview of related studies in the field. Section 3 details the research materials. Section 4 outlines the methodology employed. Section 5 presents the experimental setup, the results, and a comprehensive discussion of the study. Section 6 addresses the limitations of the current study. Finally, Section 7 concludes the paper and discusses potential directions for future work.

## 2. Related Studies

Artificial intelligence (AI) and ML have emerged as transformative tools in the field of medical diagnostics, particularly for myeloproliferative neoplasms. These sophisticated technologies offer promising solutions to longstanding challenges in myeloproliferative neoplasm diagnosis, including interobserver variability, delayed diagnosis, and the inherently subjective nature of morphological assessments. An expanding body of literature has explored the application of AI, ML, and DL methodologies in diagnosing and classifying myeloproliferative neoplasms, demonstrating their significant potential to revolutionize established clinical workflows and enhance diagnostic accuracy. In this section, we systematically categorize the existing research into three distinct domains: Deep Learning Models for Digital Pathology and Morphological Assessment, Multimodal Fusion Approaches for Enhanced Diagnostic Precision, and AI-Driven Quantitative Bone Marrow Analysis. Through this structured examination, we aim to elucidate the current landscape, evaluate methodological advances, and identify promising avenues for further investigation in this rapidly evolving field.

### 2.1. Deep Learning Models for Digital Pathology and Morphological Assessment

Recent advancements in DL have significantly enhanced the morphological assessment of myeloproliferative neoplasms, addressing longstanding challenges in subjective histopathological evaluation. Convolutional neural networks (CNNs) have emerged as a cornerstone for automated feature extraction from bone marrow biopsies (BMBx) and peripheral blood smears. For instance, Mehrtash et al. [17] employed a pre-trained CNN to analyze histomorphological features in BMBx, achieving an AUC of 0.92 for distinguishing myeloproliferative neoplasms from non-neoplastic controls. Similarly, Kimura et al. [18] demonstrated the utility of CNNs in analyzing peripheral blood specimens, achieving AUC values exceeding 0.967 for differentiating myeloproliferative neoplasm subtypes, thereby offering a less invasive diagnostic alternative. Unsupervised learning techniques have further complemented these efforts. For instance, Sirinukunwattana et al. [15] identified 9–11 distinct megakaryocyte subtypes through clustering analysis, enabling a high diagnostic AUC of 0.95 in subtyping myeloproliferative neoplasms. These approaches reduce reliance on manual annotation and mitigate interobserver variability, as demonstrated by Yusof et al. [19], whose CNN model achieved 95.3% classification accuracy for classical myeloproliferative neoplasms using hyperparameter-tuned bone marrow histopathology images.

A critical innovation in this domain is the integration of interpretability tools to bridge AI outputs with clinical trust. Srisuwananukorn et al. [20] utilized attention-based multiple instance learning (MIL) to localize diagnostically relevant regions in whole slide images (WSIs), achieving 92.3% accuracy in distinguishing pre-fibrotic myelofibrosis (prePMF) from ET. Similarly, Krichevsky et al. [21] demonstrated that MIL models could predict JAK2/CALR mutations directly from WSIs with >90% precision, underscoring AI’s ability to link morphology with molecular biology. However, these models require large, annotated datasets, a limitation partially addressed by human-in-the-loop frameworks [22] and knowledge distillation techniques [23], which transfer learning from larger histopathology datasets to smaller myeloproliferative neoplasm cohorts. Collectively, these studies highlight DL’s transformative potential in automating and standardizing morphological assessments while emphasizing the need for robust validation across diverse clinical settings.

### 2.2. Multimodal Fusion Approaches for Enhanced Diagnostic Precision

The integration of multimodal data (combining histopathological, clinical, and genomic inputs) has emerged as a pivotal strategy to overcome the limitations of single-modality AI models in myeloproliferative neoplasm diagnosis. Zhang et al. [24] pioneered this approach with their Dynamic Screening and Clinical-Enhanced Network (DSCENet), which fused WSIs with clinical indicators (e.g., blood counts, genetic mutations), improving classification AUC by 7.91% compared to image-only models. This framework not only enhanced subtype differentiation but also reduced misclassification rates between ET and prePMF by 16.89%, addressing a key diagnostic challenge. Similarly, Wang et al. [25] developed a fusion model integrating clinical parameters with DL-based bone marrow analysis, achieving superior performance (with AUC equal to 0.931 for myeloproliferative neoplasm vs. non-myeloproliferative neoplasm classification) over unimodal approaches. Their model demonstrated comparable accuracy to senior hematopathologists, particularly in distinguishing ET from prePMF with an AUC of 0.887, underscoring the clinical viability of multimodal systems.

Prognostic applications of multimodal AI have also gained traction. Asti et al. [26] merged morphological features from WSIs with genomic and clinical data to predict overall survival (OS) and leukemia-free survival (LFS), increasing Harrell’s concordance index from 0.68 to 0.87 for OS. This study further identified associations between AI-derived morphological features and mutations (e.g., SF3B1, JAK2), bridging histology with molecular pathogenesis. However, harmonizing heterogeneous data types remains a challenge, necessitating dynamic feature selection mechanisms, as exemplified by DSCENet’s screening module. Explainability tools, such as SHAP analysis [26] and attention heatmaps [20], have further enhanced clinical translatability by elucidating model decision-making processes. While these advances are promising, the field requires standardized protocols for data integration and prospective validation to ensure reproducibility across institutions.

### 2.3. AI-Driven Quantitative Bone Marrow Analysis

Quantitative AI platforms have revolutionized bone marrow analysis by replacing semi-quantitative grading systems with objective, reproducible metrics. Yu et al.  [27] developed a neural network-based platform utilizing U2-Net and ResNet to quantify cellularity, myeloid-to-erythroid (M: E) ratios, and fibrosis severity, achieving segmentation accuracy of 0.9 and AUCs up to 0.98 for myeloproliferative neoplasm subtype differentiation. Their bone marrow classification model outperformed clinical models in most cases, highlighting the diagnostic value of AI-driven histomorphometric analysis. Similarly, Ryou et al. [28] introduced the Continuous Indexing of Fibrosis (CIF). This ranking-CNN model quantified reticulin fibrosis with high precision (measured by an AUC of 0.94 for ET vs. prePMF discrimination). This approach identified ET patients at risk of progression to post-ET myelofibrosis with an AUC of 0.77, demonstrating AI’s prognostic utility. Unsupervised techniques have further advanced quantitative assessments. For instance, the systematic review by Elsayed et al. [29] highlighted principal component analysis (PCA) and DBSCAN for automated megakaryocyte analysis, enabling subtype categorization based on histotopographical features.

Sirinukunwattana et al. [15] expanded this by correlating AI-identified megakaryocyte subtypes with driver mutations (e.g., CALR), offering a bridge between morphology and genomics. These tools address the subjectivity inherent in traditional methods, aligning with WHO guidelines that prioritize bone marrow histopathology as the diagnostic gold standard [19]. Nevertheless, challenges persist in standardizing quantitative thresholds across laboratories and integrating these tools into clinical workflows. Future efforts must focus on multi-center validation and the development of user-friendly interfaces to facilitate adoption.

### 2.4. Research Gaps

The literature underscores AI’s transformative role in myeloproliferative neoplasm diagnosis through three interconnected paradigms: DL for morphological assessment, multimodal data fusion, and quantitative bone marrow analysis. DL models have demonstrated exceptional accuracy in automating feature extraction and reducing diagnostic variability. At the same time, multimodal frameworks utilize complementary data sources to resolve ambiguities in subtype classification. Quantitative AI platforms, meanwhile, provide objective metrics that enhance reproducibility and prognostic stratification. Despite these advances, challenges such as data scarcity, model interpretability, and clinical integration remain. Future research should prioritize prospective validation, interoperability standards, and the development of explainable AI tools to translate these innovations into routine practice, ultimately advancing personalized management for myeloproliferative neoplasm patients.

While previous investigations have explored the utility of AI in diagnosing and classifying myeloproliferative neoplasms, several gaps remain. First, existing models often focus on specific tasks, such as megakaryocyte analysis or cellularity quantification, leaving opportunities for developing unified frameworks that address multiple diagnostic challenges simultaneously. Second, few studies have utilized state-of-the-art approaches for feature extraction and classification, which could increase the precision and robustness of myeloproliferative neoplasm diagnostics. Advanced techniques remain underexplored in this domain despite their proven efficacy in other medical imaging applications. Third, fewer studies have focused on this problem overall, reflecting the limited attention given to rare diseases such as myeloproliferative neoplasms compared with more prevalent conditions. Addressing these gaps will be crucial for advancing AI-driven solutions in myeloproliferative neoplasm diagnostics and management.

## 3. Materials

The study design for the Histopathology Imagery Dataset of Ph-Negative Myeloproliferative Neoplasm [30] was guided by rigorous ethical and scientific standards, as outlined by the original creators. Ethical approval for the dataset was obtained from the Ministry of Health, Malaysia, under reference number NMRR-18-4023-42507(IIR), ensuring compliance with national and institutional guidelines. The dataset was anonymized to protect patient confidentiality, with no personal details included, and verbal consent was documented by attending physicians during routine clinic visits. We, as users, adhered to these ethical principles and ensured that all analyses respected the privacy and integrity of the data.

**Figure 1:**
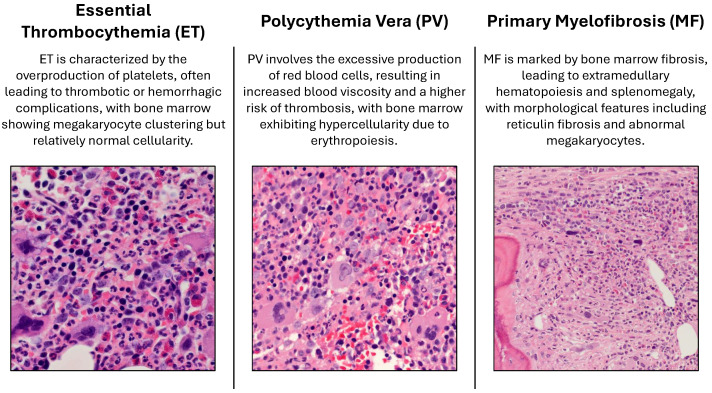
Samples from the utilized dataset for the Histopathology Imagery Dataset of Ph-Negative Myeloproliferative Neoplasm [30].

The dataset shown in Figure 1 comprises 300 high-resolution BMT images captured via an Olympus BX41 (manufactured by Olympus Corporation, a Japanese company, Tokyo Japan) dual-head microscope with ×10, ×20, and ×40 magnifications. These images focus on three major Ph-negative myeloproliferative neoplasm subtypes: ET, MF, and PV. Bone marrow morphology is critical for distinguishing between these subtypes, with each exhibiting unique histopathological features. For example, PV is characterized by increased erythropoiesis, ET by megakaryocyte clustering, and MF by reticulin fibrosis and osteosclerosis.

Patient selection for the dataset was based on strict inclusion and exclusion criteria, as defined by the original creators. Only patients diagnosed with Ph-negative myeloproliferative neoplasms were included, with equal representation across the three subtypes (100 images each for ET, PV, and MF). Imaging techniques were standardized to ensure consistency, and the dataset was organized into categories on the basis of myeloproliferative neoplasm subtype and imaging parameters. The dataset is accessible through the Mendeley v1 Data repository https://data.mendeley.com/datasets/8mds4wpch3/1 (accessed on 1 March 2024).

## 4. Methods

In this section, we describe the methodology adopted for the classification of Ph-negative myeloproliferative neoplasms. The proposed framework integrates both handcrafted and automatic feature extraction techniques to capture morphological, textural, and spatial characteristics from histopathology images. The process begins with image preprocessing. Raw RGB images are converted to the XYZ color space to ensure device-independent and perceptually aligned representation. This step facilitates normalization and enhances compatibility with advanced computational tools.

Following preprocessing, nuclei segmentation is performed via a pretrained StarDist 2D model [31,32]. The normalized images are segmented to identify individual nuclei, which serve as regions of interest for feature extraction. Handcrafted features are then computed for each nucleus, including first-order statistics (FOS), gray-level co-occurrence matrix (GLCM), gray-level run-length matrix (GLRLM), and gray-level size zone matrix (GLSZM) [33,34]. These features capture diverse aspects of nuclear morphology and texture, such as the pixel intensity distribution, spatial relationships, run-length patterns, and zone-based granularity.

To complement handcrafted features, automatic feature extraction is performed via H-optimus-0, a state-of-the-art vision transformer trained on a large dataset of hematoxylin and eosin (H&E)-stained whole-slide images [35]. Tiles extracted from the histopathology images are resized to a fixed resolution of 224×224 pixels and passed through the H-optimus-0 model to generate high-dimensional embeddings. These embeddings capture intricate histopathological patterns and are used as inputs for downstream classification tasks.

**Figure 2:**
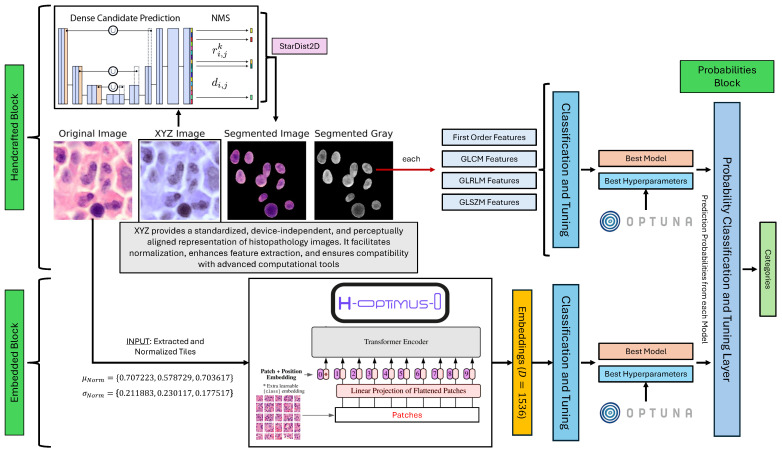
Graphical presentation of the suggested approach.

The extracted features, both handcrafted and automatic, are fed into machine-learning models for classification and tuning. Hyperparameter optimization is performed to identify the best-performing model and configuration. The framework’s graphical representation is illustrated in Figure 2, highlighting the key steps involved in the proposed approach.

### 4.1. Handcrafted Feature Extraction from Nuclei

To address the challenge of classifying Ph-negative myeloproliferative neoplasms, we employed a systematic approach for handcrafted feature extraction from nuclei in histopathology images. This process involves multiple steps, including image preprocessing, nuclei segmentation, and the computation of statistical, textural, and spatial features. These features are critical for distinguishing between myeloproliferative neoplasm subtypes such as ET, PV, and MF.

The first step in our pipeline is the segmentation of nuclei via a pretrained StarDist 2D model  [36,37,38]. Given an input image $I\in R^{H\times W\times C}$, where *H*, *W*, and *C* represent the height, width, and number of channels, respectively, the image is normalized to improve segmentation performance. The normalized image $I_{\mathrm{norm}}$ is then passed to the StarDist model to generate labeled regions.

Once the nuclei are segmented, we extract handcrafted features for each nucleus. The features include first-order statistical measures, gray-level co-occurrence matrix (GLCM) features [39], gray-level run-length matrix (GLRLM) features [40], and gray-level size zone matrix (GLSZM) features [41]. For instance, the GLCM is computed for each nucleus at various distances d∈D and angles θ∈Θ, where D={1,2,3} and $\Theta =\{0^{\circ },45^{\circ },90^{\circ },135^{\circ }\}$. The GLCM for a given distance *d* and angle θ is defined as in Equation (1), where 1(·) is the indicator function. From the GLCM, texture features such as contrast, correlation, and energy are derived via Equation (2).

(1) $$\mathrm{GLCM}\left(d,\theta \right)=\sum_{i,j}1\left(I\left(x,y\right)=i\wedge I\left(x+d\times \cos\theta ,y+d\times \sin\theta \right)=j\right)$$

(2) $$\begin{array}{cc} \mathrm{Contrast} & =\sum_{i,j}{\left(i-j\right)}^{2}\times \mathrm{GLCM}\left(i,j\right)\\ \mathrm{Correlation} & =\frac{\sum_{i,j}\left(i-\mu_{i}\right)\times \left(j-\mu_{j}\right)\times \mathrm{GLCM}\left(i,j\right)}{\sigma_{i}\times \sigma_{j}}\\ \mathrm{Energy} & =\sum_{i,j}\mathrm{GLCM}{\left(i,j\right)}^{2} \end{array}$$

Similarly, GLRLM features are computed to capture run-length patterns within the nucleus. For a given angle θ, the GLRLM is defined as in Equation (3), where *r* represents the length of consecutive pixels with the same intensity. Features such as short-run emphasis (SRE) and long-run emphasis (LRE) are then calculated via Equation (4).

(3) $$\mathrm{GLRLM}\left(\theta \right)=\sum_{r}1\left(\mathrm{run}\;\mathrm{length}=r\wedge \mathrm{angle}=\theta \right)$$

(4) $$\begin{array}{cc} \mathrm{SRE} & =\frac{\sum_{r}\frac{\mathrm{GLRLM}(r,\theta )}{r^{2}}}{\sum_{r}\mathrm{GLRLM}\left(r,\theta \right)}\\ \mathrm{LRE} & =\frac{\sum_{r}r^{2}\times \mathrm{GLRLM}\left(r,\theta \right)}{\sum_{r}\mathrm{GLRLM}\left(r,\theta \right)} \end{array}$$

#### Hypotheses and Rationale for Handcrafted Feature Extraction

The hypothesis behind using handcrafted feature extraction techniques such as FOS is that these features can effectively capture the morphological, textural, and spatial characteristics of nuclei in BMT images. These characteristics are critical for distinguishing between Ph-negative myeloproliferative neoplasm subtypes.

First-order statistical features describe the distribution of pixel intensities within a nucleus. Metrics such as the mean, variance, skewness, and kurtosis provide insights into the overall brightness, heterogeneity, and shape of the nucleus. These features are beneficial for identifying subtle differences in nuclear morphology, which are often indicative of specific myeloproliferative neoplasm subtypes. For example, increased heterogeneity in pixel intensity may correlate with abnormal cell proliferation patterns observed in MF.

The GLCM captures second-order texture features by analyzing the spatial relationships between pairs of pixels at specified distances and angles. Features derived from the GLCM, such as contrast, correlation, energy, and homogeneity, quantify textural patterns such as smoothness, coarseness, and regularity. The use of multiple distances (d∈{1,2,3}) and angles ($\theta \in \{0^{\circ },45^{\circ },90^{\circ },135^{\circ }\}$) ensures that the model captures directional and scale-dependent variations in texture. This is crucial for differentiating between myeloproliferative neoplasm subtypes. ET, PV, and MF exhibit distinct textural patterns due to varying degrees of fibrosis, clustering, and hypercellularity.

GLRLM focuses on run-length patterns, which measure the length of consecutive pixels with similar intensities along a specific direction. Features such as SRE and LRE highlight the presence of fine or coarse structures within the nucleus. These features are particularly relevant for myeloproliferative neoplasm classification because they can reveal differences in nuclear organization, such as the elongated or clustered megakaryocytes characteristic of ET or the dense fibrotic regions observed in MF.

The GLSZM quantifies the size and distribution of homogeneous zones within the nucleus. Features such as small zone emphasis (SZE) and large zone emphasis (LZE) provide information about the granularity and compactness of nuclear regions. These metrics are valuable for capturing the irregular shapes and sizes of nuclei in myeloproliferative neoplasms, which are influenced by factors such as fibrosis and dysplasia. The use of different connectivity values (C∈{4,8}) allows for robust characterization of zone boundaries, ensuring that both tightly packed and loosely connected regions are accurately represented.

The variability in distances, angles, and connectivity settings is essential for capturing the multiscale and multidirectional nature of nuclear features in BMT images. Different myeloproliferative neoplasm subtypes exhibit unique morphological and textural patterns that may manifest at varying scales and orientations. For example, shorter distances emphasize fine-grained textures, whereas longer distances capture broader structural patterns. Analyzing multiple angles accounts for the anisotropic nature of tissue structures, enabling the detection of directional biases in texture and morphology. Varying connectivity settings (4-connected vs. 8-connected) allow for flexible zone definitions, accommodating both strict and relaxed interpretations of nuclear boundaries.

### 4.2. Automatic Feature Extraction from Tiles Using H-optimus-0

To extract meaningful features from histopathology tiles, we utilized H-optimus-0, an open-source foundation model designed explicitly for computational pathology. H-optimus-0 is a state-of-the-art vision transformer trained on a vast dataset of over 500,000 H&E-stained whole-slide images, enabling it to capture highly diverse and representative histological patterns [42]. The model architecture and training framework make it particularly well suited for extracting tile-level features that can be used in downstream tasks such as classification and segmentation.

The backbone of H-optimus-0 is based on a ViT with a g/14 architecture, consisting of 40 transformer blocks. Each block contains 24 attention heads, and the embedding dimension is set to 1536. This large-scale architecture allows the model to learn intricate patterns in histopathology tiles. Given a tile $T\in R^{H\times W\times C}$, where *H*, *W*, and *C* represent the height, width, and number of channels (typically 3 for RGB), the feature extraction process can be described as follows: First, each tile is resized to a fixed resolution of 224×224 pixels to ensure compatibility with the model’s input requirements.

Next, the preprocessed tile is passed through the ViT backbone to generate a high-dimensional feature vector $F\in R^{D}$, where D=1536. The encoding process involves multiple layers of self-attention and feedforward networks. Within each transformer block, the self-attention mechanism computes attention weights *A* for every patch in the tile. For a given query *Q*, key *K*, and value *V*, the attention weights are calculated via Equation (5), where $d_{k}$ is the dimensionality of the keys. These weights are then used to compute a weighted sum of the values.

(5) $$A=\mathrm{Softmax}\left(\frac{Q\times K^{T}}{\sqrt{d_{k}}}\right)$$

H-optimus-0 offers several advantages for feature extraction in histopathology: (1) Scalability: The model is trained on one of the largest histopathology datasets, ensuring robustness across diverse tissue types. (2) Self-Supervised Learning: By using self-supervised learning frameworks, H-optimus-0 captures rich, unsupervised representations without requiring extensive labeled data. (3) Transferability: The extracted features generalize well to unseen datasets, making H-optimus-0 suitable for applications such as myeloproliferative neoplasm classification. By integrating H-optimus-0 into our methodology, we achieve a robust and efficient pipeline for extracting and utilizing histopathological features, paving the way for accurate myeloproliferative neoplasm subtype classification.

### 4.3. Rationale for Combining Handcrafted and Automatic Feature Extraction

The integration of both handcrafted and automatic feature extraction methods is motivated by their complementary strengths in capturing diverse characteristics of histopathological images. Handcrafted features, such as the FOS, GLCM, GLRLM, and GLSZM, are explicitly designed to encode domain-specific knowledge about nuclear morphology, texture, and spatial relationships. These features provide interpretable insights into the underlying biological processes. They are particularly valuable for tasks requiring explainability, such as distinguishing between subtypes of Ph-negative myeloproliferative neoplasms. For example, the GLCM captures second-order textural patterns that reflect the smoothness or coarseness of tissue structures. In contrast, the GLSZM quantifies the granularity and compactness of nuclear regions, which are critical for identifying irregularities associated with myeloproliferative neoplasm subtypes.

On the other hand, automatic feature extraction via foundation models such as H-optimus-0 uses deep learning architectures to learn high-dimensional representations directly from data. These models excel at capturing complex, hierarchical patterns in histopathology tiles that handcrafted features may not explicitly encode. The vision transformer backbone of H-optimus-0, with its 40 transformer blocks and 24 attention heads, enables the model to generalize across diverse tissue types and extract features that are robust to variations in scale, orientation, and staining. This capability is particularly advantageous for handling the heterogeneity inherent in Ph-negative myeloproliferative neoplasm datasets, where morphological and textural patterns can vary significantly between patients and subtypes.

By combining handcrafted and automatic features, we aim to achieve a balanced approach that utilizes the interpretability of handcrafted features and the representational power of automatic features. Handcrafted features provide a strong baseline for capturing known morphological and textural characteristics. In contrast, automatic features offer the potential to uncover latent patterns that may not be immediately apparent to human experts.

### 4.4. Training and Optimization

The training and optimization process involves the systematic tuning of machine learning models to achieve optimal performance in classifying Ph-negative myeloproliferative neoplasms. The pipeline begins with data preprocessing, where features extracted from histopathology images are scaled and selected via various techniques. Feature scaling methods include standardization, min–max normalization, and robust scaling, whereas feature selection techniques such as recursive feature elimination (RFE), principal component analysis (PCA), and mutual information (MI) are employed to reduce dimensionality and enhance model interpretability.

Hyperparameter optimization is performed via the Optuna framework [43], which employs a tree-structured Parzen estimator (TPE) sampler to maximize the weighted average metric. The optimization process iterates over multiple trials, each involving a random seed for reproducibility. During each trial, the dataset is split into training and testing subsets, with the testing subset comprising 15% of the data. The objective function evaluates different combinations of classifiers, scalers, and feature selection techniques, recording metrics such as accuracy, precision, recall, F1 score, and specificity for each configuration.

The best-performing model is identified on the basis of the highest weighted average metric across all trials. The results are logged into a history file, which includes detailed records of each trial’s parameters and performance metrics. This iterative approach ensures that the final model is both robust and generalizable.

In the first block of classification and tuning, we focused on embedded features extracted via H-optimus-0, which captures rich, unsupervised representations of the tissue patterns, enabling effective downstream classification tasks.

Through hyperparameter optimization, the multilayer perceptron (MLP) classifier emerged as the best-performing model for this feature type. The MLP was able to utilize the high-dimensional embeddings generated by H-optimus-0, achieving superior performance in distinguishing between the ET, PV, and MF subtypes. The optimization process involved feature scaling via robust normalization and feature selection via MI (for 25% of the features), further enhancing the model’s ability to generalize across diverse tissue types.

The second block of classification and tuning focused on handcrafted features that included first- and second-order statistical measures critical for differentiating between myeloproliferative neoplasm subtypes. Similar to the embedded features, the MLP classifier demonstrated the best performance for handcrafted features. The optimization process highlighted the importance of feature scaling via standard scaling and feature selection via PCA (for 70% of the features), which helped mitigate noise and redundancy in the hand-crafted feature set.

In the third block, we explored the use of concatenated probabilities derived from the predictions of the two preceding models (embedded features and hand-crafted features). This approach aims to combine the strengths of both feature types, utilizing the complementary information they provide. For this task, the stochastic gradient descent (SGD) classifier emerged as the best-performing model. Feature scaling via min–max normalization and feature selection via RFE (for 85% of the features) further enhanced the model’s performance, ensuring that the most informative features were prioritized during training.

### 4.5. Performance Measures

To evaluate the performance of the proposed framework, we employ a comprehensive set of metrics derived from the confusion matrix. These metrics include the following:

- Accuracy: The proportion of correctly classified instances out of the total instances;
- Precision: The ratio of true positives to the sum of true positives and false positives, indicating the model’s ability to avoid false alarms;
- Recall (Sensitivity): The ratio of true positives to the sum of true positives and false negatives, reflecting the model’s ability to identify all relevant cases;
- F1-Score: The harmonic mean of precision and recall, providing a balanced measure of the two;
- Specificity: The ratio of true negatives to the sum of true negatives and false positives, highlighting the model’s ability to identify negative cases correctly;
- Weighted average metric: A composite score that accounts for class imbalance by weighting each metric by the number of instances in each class.

### 4.6. The Proposed Framework Pseudocode

The proposed framework integrates handcrafted and automatic feature extraction with machine learning classification and hyperparameter optimization. Algorithm 1 presents the key steps.
**Algorithm 1:**The proposed framework for myeloproliferative neoplasm classification via handcrafted and automatic feature extraction.
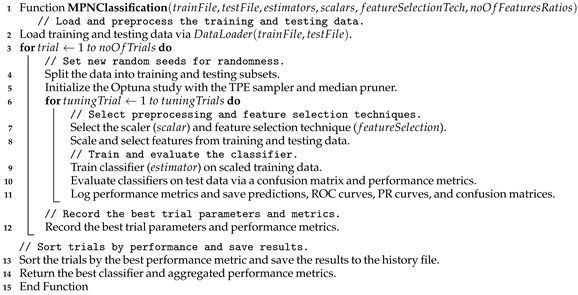


## 5. Experiments and Discussion

To systematically explore the performance of various machine learning configurations, we employed hyperparameter optimization using Optuna with the TPE algorithm. During this process, we evaluated a range of classifiers, feature scalers, and feature selection techniques to identify the best-performing combination for each feature type (handcrafted, embedded, and concatenated probabilities). The specific methods used are summarized in Table 1.

### 5.1. Experimental Results

Table 2 summarizes the performance comparison between our approach using the three blocks. The results demonstrate the effectiveness of each feature type (handcrafted, embedded, and concatenated probabilities) in classifying Ph-negative myeloproliferative neoplasms. Each block was evaluated via a range of metrics, including precision, recall, F1 score, accuracy, specificity, and an overall weighted average.

The handcrafted features, derived from nuclei segmentation and statistical analysis, achieved high performance across all the metrics, with a mean weighted average of 0.9765. This highlights the importance of morphological and textural features in distinguishing between myeloproliferative neoplasm subtypes.

The embedded features extracted via the H-optimus-0 model also performed well, achieving a mean weighted average of 0.9686. These features utilize the power of deep learning to capture complex patterns in histopathological images, providing a robust representation for classification tasks.

Finally, the concatenated probabilities, which combine predictions from both handcrafted and embedded features, achieved the highest performance, with a mean weighted average of 0.9969. This demonstrates the complementary nature of the two feature types and the potential for ensemble methods to improve classification accuracy further.

### 5.2. Discussion

The experimental results highlight the strengths and limitations of each feature type in classifying Ph-negative myeloproliferative neoplasms. These features provide interpretable insights into nuclear morphology and texture, capturing subtle differences indicative of specific myeloproliferative neoplasm subtypes, such as abnormal cell proliferation patterns in MF. However, they are computationally intensive.

Embedded features utilize pretrained vision transformers such as H-optimus-0 to capture diverse histological patterns. Their scalability and transferability make them suitable for scenarios with limited labeled data, but their lack of interpretability may hinder their clinical adoption.

Concatenated probabilities integrate handcrafted and embedded features, achieving superior performance. This underscores the value of combining multiple information sources to increase diagnostic accuracy. The use of SGD as a classifier further demonstrates the flexibility of machine learning models in handling varied input data.

### 5.3. Statistical Analysis

The statistical analysis evaluates the robustness and reliability of the classification results across three feature types: handcrafted, embedded, and concatenated probabilities. This section outlines the metrics used, the experimental setup, and the key findings.

#### 5.3.1. Performance Metrics and Statistical Tests

To assess the classification performance, we computed the mean and standard deviation of metrics such as accuracy, recall, F1 score, and specificity. One-sample *t*-tests were performed to determine whether the observed results significantly deviated from expected values (p≥0.05). Effect sizes (Cohen’s *d* and Hedges’ *g*) were calculated to quantify the magnitude of differences, providing practical insights beyond statistical significance.

Normality tests, including Shapiro–Wilk, Jarque–Bera, and Kolmogorov–Smirnov, were conducted to verify the distributional assumptions of the data. These tests guided the choice of parametric or nonparametric methods for further analysis.

#### 5.3.2. Experimental Setup

The analysis was based on performance metrics obtained from 10 independent runs for each feature type. This approach ensures robustness while accounting for variability in classification outcomes. For each run, metrics were computed, and their means and standard deviations were calculated. The use of 10 runs aligns with common practices in machine learning experiments, balancing statistical rigor with computational efficiency.

#### 5.3.3. Key Findings

Handcrafted Features: The weighted average metric achieved a mean of 0.9765 and a standard deviation of 0.0010. One-sample *t*-tests revealed no significant deviations (p≥0.05), with negligible effect sizes ($Cohen^{\prime }s\;d=0.0$, $Hedges^{\prime }\;g=0.0$). Normality tests indicated non-normal distributions (Shapiro–Wilkp≤0.05, Jarque–Berap=0.027, $\mathrm{Kolmogorov}–\mathrm{Smirnov}\;p=3.89\times 10^{-12}$).

Embedded Features: The weighted average metric had a mean of 0.9686 and a standard deviation of 0.0055. Similar to handcrafted features, no significant deviations were found (p≥0.05), and effect sizes were negligible. While Shapiro–Wilk and Jarque–Bera tests suggested normality (p>0.05), the Kolmogorov–Smirnov test indicated deviations ($p=5.40\times 10^{-12}$).

Concatenated Probabilities: Concatenated probabilities achieved the highest mean weighted average of 0.9969, with a standard deviation of 0.0005. Again, no significant deviations were observed (p≥0.05), and effect sizes were negligible. Normality tests were mixed: Shapiro–Wilk tests indicated non-normality (p≤0.05), Jarque–Bera tests suggested normality (p=0.557), and Kolmogorov–Smirnov tests confirmed deviations ($p=2.32\times 10^{-12}$).

#### 5.3.4. Insights

**Figure 3:**
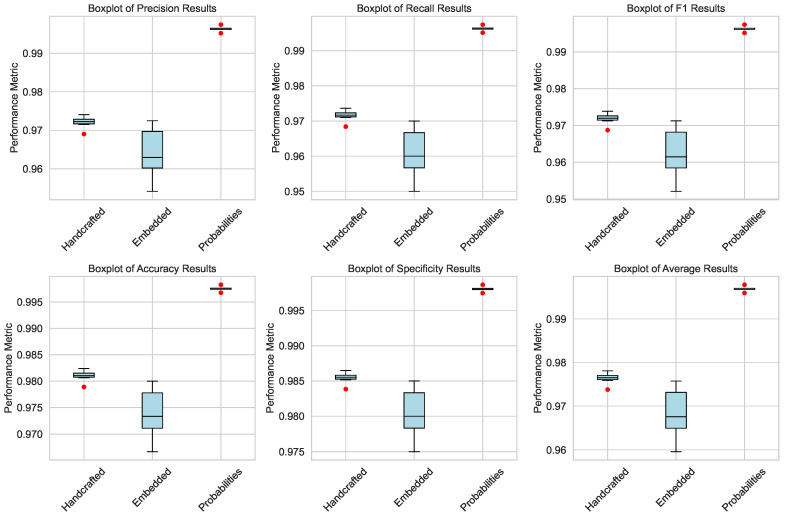
Boxplot representation of performance metrics across different feature types, illustrating variability and central tendencies.

**Figure 4:**
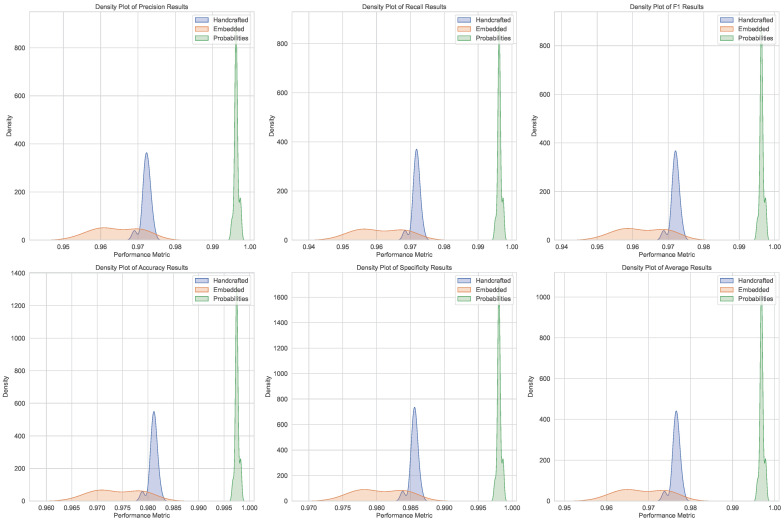
Density plot of performance metrics, highlighting the distribution patterns for handcrafted, embedded, and concatenated probability features.

The statistical analysis confirms the reliability of the classification results, with consistent metrics and negligible effect sizes. However, challenges in assuming normal distributions for handcrafted and concatenated probability features (as shown in Figure 3 and Figure 4) highlight the need for both parametric and nonparametric methods in future analyses.

### 5.4. Potential Clinical Applications and Integration Challenges

The proposed dual-feature framework has significant potential for translation into clinical practice, offering several advantages over traditional diagnostic methods for Philadelphia chromosome-negative (Ph-negative) myeloproliferative neoplasms. By integrating handcrafted features that capture interpretable morphological and textural characteristics with automatic features derived from state-of-the-art vision transformers, the framework provides a comprehensive and objective approach to myeloproliferative neoplasm classification. This combination not only enhances diagnostic accuracy but also addresses critical challenges in current clinical workflows, such as interobserver variability and delayed diagnosis.

One of the primary clinical applications of our model is in the automated analysis of bone marrow biopsy images. The framework can assist pathologists by providing rapid and reproducible predictions of myeloproliferative neoplasm subtypes, reducing the time required for manual evaluation and improving diagnostic consistency. For instance, the ability to distinguish between essential thrombocythemia, polycythemia vera, and primary myelofibrosis with high accuracy (weighted average of 0.9969 for concatenated probabilities) can support early intervention and personalized treatment planning. Additionally, the model’s robust performance across diverse metrics (e.g., precision, recall, F1 score) underscores its reliability in identifying subtle differences in nuclear morphology and tissue patterns.

Beyond diagnosis, the framework can also contribute to prognostic stratification and treatment monitoring. By quantifying histopathological features such as fibrosis severity, cellularity, and megakaryocyte clustering, the model can help predict disease progression and guide therapeutic decisions. For example, patients with post-ET myelofibrosis could be identified earlier through the model’s ability to detect reticulin fibrosis and abnormal megakaryocytes, enabling timely interventions to manage complications such as splenomegaly and extramedullary hematopoiesis.

Despite these promising applications, several challenges must be addressed to facilitate the integration of our model into real-world clinical settings. First, clinician acceptance remains a critical barrier, particularly for deep learning-based embeddings that lack interpretability. To overcome this, future iterations of the framework could incorporate XAI techniques, such as attention heatmaps or SHAP analysis, to provide insights into the model’s decision-making process. These tools would enhance transparency and build trust among clinicians, encouraging broader adoption.

Second, the scalability and accessibility of the framework need to be considered. While the model achieves superior performance using high-resolution histopathology images, its deployment in resource-limited settings may be hindered by the lack of advanced microscopy equipment or digital pathology infrastructure. To address this, lightweight versions of the framework could be developed, utilizing edge computing or cloud-based solutions to enable remote access and analysis.

Finally, regulatory approval and validation are essential steps before the model can be integrated into clinical practice. Multicenter studies involving diverse patient populations and imaging conditions are needed to evaluate the generalizability of the framework and ensure compliance with regulatory standards. Collaboration with healthcare institutions and regulatory bodies will be crucial to navigate this process effectively.

## 6. Limitations

Despite its promising results, the proposed framework has limitations that warrant consideration. First, the dataset used in this study, while rigorously curated, is relatively small and may not fully represent the diversity of Ph-negative myeloproliferative neoplasms across different populations. Second, the reliance on high-resolution histopathology images poses challenges for institutions with limited access to advanced microscopy equipment or digital pathology infrastructure. Finally, the lack of interpretability in deep learning-based embeddings remains a barrier to widespread clinical adoption, as clinicians often require transparent and explainable models to trust AI-driven predictions.

## 7. Conclusions and Future Work

This study demonstrates the potential of combining handcrafted and automatic feature extraction techniques to classify Ph-negative myeloproliferative neoplasms accurately. By utilizing morphological, textural, and spatial features alongside high-dimensional embeddings from H-optimus-0, the proposed framework achieves superior performance compared with individual feature types. Statistical analysis confirms the robustness of the results, highlighting the consistency and reliability of the classification metrics. These findings underscore the value of integrating multiple information sources to increase diagnostic accuracy and provide a foundation for future research in computational pathology.

Future work should focus on addressing the identified limitations by expanding the dataset to include a broader range of patients and imaging conditions. Incorporating multicenter data would improve the generalizability of the framework and facilitate its validation across diverse clinical settings. In addition, the efforts should be directed towards optimizing the computational efficiency of the pipeline, potentially through lightweight deep learning architectures or edge computing solutions.

## Figures and Tables

**Table 1:** The experimental configurations of the current study.

Configuration	Value(s)
StartDist2D	${NMS}_{Threshold}=0.1$ and $p_{Threshold}=0.65$
Angle	$\Theta =\{0^{\circ },45^{\circ },90^{\circ },135^{\circ }\}$
Distance	D={1,2,3}
Connectivity	C={4,8}
Tile Shape	(256,256)
Hyperparameters Optimization	Optuna
Tuning Algorithm	TPE
Feature Scalers	Standardization, min–max normalization, robust scaling, max–absolute normalization
Feature Selection	Recursive feature elimination, principal component analysis, and mutual information
Classifiers	All suitable estimators from Scikit-Learn package such as Logistic Regression, Support Vector Machine (SVM), Random Forest, Gradient Boosting (e.g., XGBoost, LightGBM), k-Nearest Neighbors (KNN), Stochastic Gradient Descent (SGD), Multi-layer Perceptron (MLP), Gaussian Naive Bayes, and Decision Trees

**Table 2:** Performance comparison of related studies and our suggested approach.

Type	Precision		Recall		F1		Accuracy		Specificity		Average	
	Mean	Std.	Mean	Std.	Mean	Std.	Mean	Std.	Mean	Std.	Mean	Std.
Handcrafted	0.9722	0.0012	0.9717	0.0012	0.9720	0.0012	0.9811	0.0008	0.9855	0.0006	0.9765	0.0010
Embedded	0.9644	0.0060	0.9611	0.0069	0.9628	0.0064	0.9741	0.0046	0.9806	0.0034	0.9686	0.0055
Probabilities	0.9963	0.0005	0.9963	0.0005	0.9963	0.0005	0.9976	0.0004	0.9981	0.0003	0.9969	0.0005

## Data Availability

The dataset is accessible through the Mendeley Data repository: https://data.mendeley.com/datasets/8mds4wpch3/1. (accessed on 1 March 2024).
